# Response: Sharpe, Goldsmith and Chalder fail to restore confidence in the PACE trial findings

**DOI:** 10.1186/s40359-019-0296-x

**Published:** 2019-03-26

**Authors:** Carolyn E. Wilshire, Tom Kindlon

**Affiliations:** 1 School of Psychology, Victoria University of Wellington, P.O. Box 600, Wellington, New Zealand; 2 Irish ME/CFS Association, Dublin, Republic of Ireland

**Keywords:** Clinical trial, Chronic fatigue syndrome, Myalgic encephalomyelitis, Graded exercise therapy, Cognitive behavioral therapy, Methodology

## Abstract

In a recent paper, we argued that the conclusions of the PACE trial of chronic fatigue syndrome are problematic because the pre-registered protocol was not adhered to. We showed that when the originally specific outcomes and analyses are used, the evidence for the effectiveness of CBT and graded exercise therapy is weak. In a companion paper to this article, Sharpe, Goldsmith and Chalder dismiss the concerns we raised and maintain that the original conclusions are robust. In this rejoinder, we clarify one misconception in their commentary, and address seven additional arguments they raise in defence of their conclusions. We conclude that none of these arguments is sufficient to justify digressing from the pre-registered trial protocol. Specifically, the PACE authors view the trial protocol as a preliminary plan, subject to honing and improvement as time progresses, whereas we view it as a contract that should not be broken except in extremely unusual circumstances. While the arguments presented by Sharpe and colleagues inspire some interesting reflections on the scientific process, they fail to restore confidence in the PACE trial’s conclusions.

Publications from the PACE trial reported that adding cognitive behavioural therapy (CBT) or graded exercise therapy (GET) to basic medical care significantly improved self-rated fatigue and physical function in a cohort of patients with chronic fatigue syndrome, and also increased the likelihood of recovery. [1, 2] However, the published analyses did not adhere to the pre-registered trial protocol. [3] We recently reanalysed a portion of the trial data using the original pre-registered outcome measures. [4] We found that the evidence for the beneficial effects of CBT or GET was weak, and did not reach the threshold of statistical significance after correcting for the number of originally planned comparisons. Neither treatment significantly increased the rate of recovery. We also raised concerns about the trial’s heavy reliance on self-report measures, which introduces a significant source of bias when a trial is not blinded.

In their recent response to our reanalysis, Sharpe, Goldsmith and Chalder ([5]) dismiss the concerns we raised in our paper and maintain that the conclusions of the PACE trial are robust.

Before addressing their arguments, we first clarify one point. The PACE trial also examined a novel behavioural treatment, called adaptive pacing therapy (APT), which did not yield reliably greater improvement than medical care alone. Sharpe et al. appear to believe that we excluded the APT trial arm from our analyses. This was not the case. The omnibus analyses reported in our paper *always included the APT arm*. We simply chose not to comment any further on those results, because they were not at issue. Our approach is stated clearly in the Methods section:

“All omnibus analyses … included the adaptive pacing therapy group, because it forms part of the trial design.” ([4], p. 4).

Having addressed this misunderstanding, let us consider Sharpe et al.’s seven remaining arguments in defence of the trial’s original conclusions. [5] Many of these were explicitly addressed in our original paper [4], and where this is the case, we refer to the relevant section.

## Argument 1: That the changes to the outcome measures were insubstantial, and there is no reason to prefer the original measures to the modified ones

The pre-registered primary outcome measure was whether participants met the specified threshold for improvement in self-reported fatigue and physical function. Several years after trial preregistration, the investigators decided this measure was “hard to interpret” ([6], p. 25). They replaced it with the continuous scores generated by the two original self-report scales, and they also modified the scoring method for the fatigue scale. [5] In addition, they substantially loosened the definition of recovery used in secondary analyses, making it much easier for patients to qualify as recovered. [2] These changes are clearly not insubstantial. Further, as we showed in our paper, all of them resulted in more successful outcomes than would have been obtained using the pre-registered measures. [4]

Sharpe et al. argue that the pre-specified outcome measures are “no more valid” than the modified ones ([5], p. 4). This argument is puzzling. The purpose of pre-registration is to prevent researchers from altering their outcome measures in ways that favour their hypotheses, after they have begun to observe the trial’s progress. Therefore, all other things being equal, measures that are stipulated ahead of time *will always* trump those formulated after the fact. Sharpe et al. offer the justification that changing the scoring method for the fatigue scale made it “more accurate and sensitive to change” ([5], p. 1). However, they provide no evidence to support this claim.

The concept of pre-registration forms the cornerstone of a good clinical trial, and this is the reason it is so vital to get good statistical advice *before* the trial begins, especially on matters such as the sensitivity, validity and interpretability of the primary outcome measures. Of course, it is perfectly acceptable to report additional, exploratory analyses that come to mind at a later date, but these should not *replace* the originally-specified measures.

An additional reason to prefer the pre-registered primary outcomes is that they formed the basis of the power analyses conducted to determine sample size. Given that the trial was estimated to be sufficiently well-powered to detect effects on a binary outcome measure, the failure to observe such effects reliably is of central interest, and should have been highlighted in the trial publications.

With regard to the recovery measure, we previously addressed all of Sharpe et al.’s justifications for altering these in our original paper, and see no need to repeat those arguments here (see [4] p. 8, see also [7, 8]). To summarise, Sharpe et al. “prefer” their modified definition because it generates similar rates of recovery to previous studies, and is also more consistent with “our clinical experience” ([5], p. 6). Clearly, it is not appropriate to loosen the definition of recovery simply because things did not go as expected based on previous studies. Researchers need to be open to the possibility that their results may not align with previous findings, nor with their own preconceptions. That is the whole point of a trial. Otherwise, the enterprise ceases to be genuinely informative, and becomes an exercise in belief confirmation.

## Argument 2: That the changes to the outcome measures were acceptable because certain procedures were followed

The various changes were fully detailed in a separate document published in 2013 ([9]), which Sharpe et al. claim was approved by the trial steering and data monitoring committees. They believe that no further justification is required.

For the reasons outlined above, pre-registered primary outcome measures have a special status in science, which is devalued if we allow researchers to alter them without strong justification. Administrative approval by a committee is simply not sufficient. In our paper, we showed that the investigators’ scientific justifications failed to stand up to careful scrutiny (see [4], pp. 7–8). And clearly, a document published in 2013 - *two years after* the primary results were reported – simply cannot be used as a replacement for the original pre-registered trial protocol.

## Argument 3: That our reanalysis was methodologically flawed

Putting aside the erroneous criticism regarding the APT arm, Sharpe and colleagues raised three further criticisms of our reanalysis. The first was that we did not adhere to “an *a priori* analysis plan” ([5], p. 1). This claim is puzzling, because of course we followed *the investigators’ own analysis plan as set out in their trial protocol* – or to be precise, we followed it as closely as was possible, given the data we had available. All our decisions were based on the best possible fit to what was stipulated in the protocol, or where no guidance was provided, we referred to other trial publications. All these decisions are fully documented in our paper ([4], p.4).

Second, Sharpe et al. criticise our method of correcting for multiple comparisons, which took into account all six planned comparisons specified in the original trial protocol. They argue that a gentler correction was more appropriate because we were primarily interested in only two comparisons. However, again, our objective was to report the results that would have been obtained *if the trial protocol had been adhered to*. Since there were six comparisons planned in that protocol, six is the appropriate number to correct for.

Sharpe et al.’s final criticism was that our analysis “only used part of the trial dataset” ([5], p. 1). It is correct that we did not have access to data for several stratification variables (e.g., centre location, therapist). However, we explored the possible impact of these omissions in our paper, concluding that it was likely to be minimal ([4], pp. 4–5). The reason our dataset was so limited was because the PACE investigators had been unwilling to share their data. The (small portion of) data we analysed was made available only after a successful application under the UK Freedom of Information Act. [10]

Sharpe et al. appear to prefer their own, unpublished analysis of the original primary outcome measures, which they conducted in 2016, shortly after they had been directed to release the relevant data. [5]. However, their method of analysis diverged in several substantive ways from the preregistration method. Our analysis is therefore to be preferred.

## Argument 4: That the absence of treatment effects at long-term follow-up is of no importance; what matters is that scores did not actually decline

A 2015 paper reported that, at long-term follow-up, there were no longer any significant differences amongst the trial arms. [11] Sharpe et al. dismiss this null result, arguing that additional, optional treatments given after the trial’s conclusion may have obscured any real treatment effects. [5] Instead, they emphasise the fact that numerical scores did not significantly decline between the trial end point and long-term follow-up. There are two problems with this reasoning. The first is that there was no evidence to support the speculation that post-trial therapy obscured genuine group differences (in our paper, we showed that the pattern of results was much the same when participants who received substantial additional post-trial therapy were excluded). Second, in a clinical trial, it is inappropriate to directly compare scores at two timepoints, especially when the number of drop-outs is large (almost a quarter of all participants) and almost certainly non-random. The only defensible conclusion here is that the small self-reported benefits of CBT and GET over the other treatment arms were no longer evident at long-term follow-up.

## Argument 5: That there is no reason to be concerned about bias associated with the trial’s reliance of self-report measures

In our paper, we argued that because the PACE trial was non-blinded, and only CBT and GET participants were told their treatments were “effective”, then any self-reported improvements are likely to be biased. Sharpe et al. believe that any such bias would be small, because: a) participants did not just give global ratings, but rather answered specific questions about their fatigue and physical function; and b) other, secondary self-report measures patterned in a similar way. They appear to be unaware that biases can be observed on a wide range of different kinds of self-report measures, including symptom-specific ones, and that they generally operate in the same direction across all types of self-report measures (see [12] for a review and metanalysis). When assessing whether self-reported measures are influenced by bias, we must examine whether they pattern in a similar way to those observed on more objective measures (e.g., estimates of physical fitness, activity levels). However, on the majority of the objective measures examined in the PACE trial, CBT and GET fared no better than the other treatment arms (for discussion, see [4] p. 10).

Sharpe and colleagues also repeat the argument that CBT and GET participants did not have higher expectations than other participants *at trial outset*. We addressed this argument in our paper, and showed that information provided *during CBT and GET* would have been likely to significantly heighten those expectations (see [4], p. 9).

## Argument 6: That the PACE trial findings are robust, because they are in line with both previous and subsequent trials

Sharpe et al. believe that because the PACE trial’s findings were consistent with other studies examining similar interventions, that this demonstrates their robustness. Of course, convergence with previous findings is not in itself proof of sound methodology. Also, these studies were subject to the same problems as the PACE trial – plus some additional ones - so it is not at all surprising that they also yielded positive outcomes. The majority were not pre-registered, were based on small samples and were problematic in various other ways (e.g. use of a passive control condition, significant loss of participants to follow-up). When we also consider that positive outcomes are more likely to be published than negative ones, then this body of evidence begins to look very problematic indeed.

## Criticisms of the trial are based on a principled objection to “psychological” explanations of chronic fatigue syndrome and are therefore invalid

Sharpe and colleagues suggest that objections to the PACE trial findings (including, presumably our critique) may stem from a principled aversion to “psychological” models of CFS. They maintain that the treatments trialled were theory-neutral, and that the trial findings do not speak to the question of illness causation.

The issue of ideological bias is an important one. But before we address it, one point needs clarification. The treatments tested in the PACE trial were *not* assumption-free. CBT and GET were explicitly based on a behavioural/deconditioning model of CFS, which assumes that there is no underlying disease process ([1]), and that the patient’s thoughts, feelings and behaviours are the primary factors maintaining the illness. The relevant therapy manuals make this position explicit. For example, the CBT participants’ manual tells them that “there is nothing to stop your body from gaining strength and fitness” ([13], p. 31). The GET manual assures patients that increasing activity will not cause any harm ([14], p.79). If PACE’s behavioural/deconditioning model of CFS proved to be unfounded, then these interventions would need to undergo substantial modification – and the trial’s conclusions would not be generalizable to these new interventions.

Turning now to ideology, the PACE trial investigators began work on the trial with the firm belief that thoughts, feelings and behaviours were the central perpetuators of CFS, and that psychological interventions could reverse the illness ([15–19]; see also [20] for discussion). In contrast, we approached our analysis from a more conservative, sceptical perspective: we considered that a false positive conclusion regarding the benefits of CBT and GET could be harmful for patients. For example, it could limit patients’ treatment options and reduce the opportunities for future research into new treatments. Readers can consider the original findings and the reanalysis in the context of these two very different perspectives and draw their own conclusions.

## Conclusion

New arguments presented by Sharpe et al. [5] inspire some interesting reflections on the scientific process, but they fail to restore confidence in the PACE trial’s original conclusions. The unjustified optimism surrounding CBT and GET – fuelled by the PACE trial publications – has almost certainly hindered the search for more effective treatments. Patients with this illness suffer terribly and they are desperate for treatments that really work. It is time to turn our attention to other approaches.

In addition, many of the issues discussed above have importance that goes well beyond CBT, GET and even chronic fatigue syndrome. In the field of psychology, there is a growing awareness that some of our scientific practices are problematic, and that serious reform is needed to improve the quality of our evidence base. [21–24] The current rejoinder touched on several central themes in this methodological debate, including the potential dangers of diverging from a preregistered protocol, the subtle biases introduced by researchers’ own beliefs and allegiances, and also the problems associated with the use of self-report measures. It is likely that the debate concerning the PACE trial will have implications reaching far beyond the illnesses and treatments under investigation.
